# Supplementary material for: Systematic Review of Smoking Cessation Interventions for Smokers Diagnosed with Cancer
Source: Int J Environ Res Public Health. 2022 Dec 18;19(24):17010. doi: 10.3390/ijerph192417010 (PMC9779002; doi:10.3390/ijerph192417010)
Supplement: Supplementary file 1 [file ijerph-19-17010-s001.zip › Supplementary File S2.pdf]

PubMed, EMBASE, CINAHL and PsycINFO

PubMed search Strategy

P

((Pulm* OR Lung OR Cervi* OR Breast OR Head OR Neck OR “Head and neck”) AND (cancer* OR Neoplasm* OR carcinoma* OR Tumor* OR Tumour* OR squamous OR oat cell*)) OR “non-small-cell lung cancer” OR “small-cell lung cancer” OR NSCLC OR SCLC OR “Cancer of Lung” OR "Lung Neoplasms"[Mesh] OR "Uterine Cervical Neoplasms"[Mesh] OR "Breast Neoplasms"[Mesh] OR "Head and Neck Neoplasms"[Mesh] OR "Carcinoma, Squamous Cell"[Mesh] OR "Small Cell Lung Carcinoma"[Mesh]

AND

Smoker* OR smoked OR "Smokers"[Mesh]

And

I

((smoking) AND (quit* OR Give OR Givi* OR stop* OR Ceas* OR Abstain* OR cut OR Cutt* OR Reduc*)) OR “tobacco use cessation” OR “tobacco cessation” OR “smoking cessation” OR “nicotine gum” OR “Nicotine patch” OR e-cigarette OR “electronic cigarette” OR “Nicotine nasal spray” OR “Nicotine lozenge” OR "Smoking Cessation Agents"[Mesh] OR "Smoking Cessation"[Mesh] OR "Tobacco Use Cessation Devices"[Mesh] OR "Tobacco Use Cessation"[Mesh] OR "Smoking Reduction"[Mesh] OR "Nicotine Chewing Gum"[Mesh] OR "Electronic Nicotine Delivery Systems"[Mesh]

Embase

P

((Pulm* OR Lung OR Cervi* OR Breast OR Head OR Neck OR “Head and neck”) AND (cancer* OR Neoplasm* OR carcinoma* OR Tumor* OR Tumour* OR squamous OR oat cell*)) OR “non-small-cell lung cancer” OR “small-cell lung cancer” OR NSCLC OR SCLC OR “Cancer of Lung” OR 'uterine cervix cancer'/exp OR 'breast cancer'/exp OR 'lung cancer'/exp OR 'head and neck tumor'/exp

AND

Smoker* OR smoked OR 'current smoker'/exp

And

I

((smoking) AND (quit* OR Give OR Givi* OR stop* OR Ceas* OR Abstain* OR cut OR Cutt* OR Reduc*)) OR “tobacco use cessation” OR “tobacco cessation” OR “smoking cessation” OR “nicotine gum” OR “Nicotine patch” OR e-cigarette OR “electronic cigarette” OR “Nicotine nasal spray” OR “Nicotine lozenge” OR 'smoking cessation'/exp OR 'smoking cessation agent'/exp OR 'nicotine gum'/exp OR 'smoking reduction'/exp OR 'nicotine patch'/exp OR 'electronic cigarette'/exp OR 'nicotine nasal spray'/exp OR 'nicotine lozenge'/exp

CINAHL

P

((Pulm* OR Lung OR Cervi* OR Breast OR Head OR Neck OR “Head and neck”) AND (cancer* OR Neoplasm* OR carcinoma* OR Tumor* OR Tumour* OR squamous OR oat cell*)) OR “non-small-cell lung cancer” OR “small-cell lung cancer” OR NSCLC OR SCLC OR “Cancer of Lung” OR (MH "Lung Neoplasms+") OR (MH "Breast Neoplasms+") OR (MH "Cervix Neoplasms+") OR (MH "Head and Neck Neoplasms+") OR (MH "Carcinoma, Squamous Cell+") OR (MH "Carcinoma, Non-Small-Cell Lung") OR (MH "Carcinoma, Small Cell")

AND

Smoker* OR smoked

And

I

((smoking) AND (quit* OR Give OR Givi* OR stop* OR Ceas* OR Abstain* OR cut OR Cutt* OR Reduc*)) OR “tobacco use cessation” OR “tobacco cessation” OR “smoking cessation” OR “nicotine gum” OR “Nicotine patch” OR e-cigarette OR “electronic cigarette” OR “Nicotine nasal spray” OR “Nicotine lozenge” OR (MH "Tobacco Use Cessation Products+") OR (MH "Smoking Cessation") OR (MH "Nicotine Chewing Gum")

Psycinfo

((Pulm* OR Lung OR Cervi* OR Breast OR Head OR Neck OR “Head and neck”) AND (cancer* OR Neoplasm* OR carcinoma* OR Tumor* OR Tumour* OR squamous OR oat cell*)) OR “non-small-cell lung cancer” OR “small-cell lung cancer” OR NSCLC OR SCLC OR “Cancer of Lung”

AND

Smoker* OR smoked

And

I

((smoking) AND (quit* OR Give OR Givi* OR stop* OR Ceas* OR Abstain* OR cut OR Cutt* OR Reduc*)) OR “tobacco use cessation” OR “tobacco cessation” OR “smoking cessation” OR “nicotine gum” OR “Nicotine patch” OR e-cigarette OR “electronic cigarette” OR “Nicotine nasal spray” OR “Nicotine lozenge” OR MAINSUBJECT.EXACT("Smoking Cessation") OR MAINSUBJECT.EXACT("Electronic Cigarettes")
