# Supplementary material for: Systematic Review of Smoking Cessation Interventions for Smokers Diagnosed with Cancer
Source: Int J Environ Res Public Health. 2022 Dec 18;19(24):17010. doi: 10.3390/ijerph192417010 (PMC9779002; doi:10.3390/ijerph192417010)
Supplement: Supplementary file 1 [file ijerph-19-17010-s001.zip › Supplementary File S3.pdf]

Supplemental Table Quality Review of Studies

Study	Study Design	Screening questions		Qualitative					Quantitative																			
									Randomised controlled trial					Non-randomised controlled trial					Quantitative descriptive					Mixed methods				
		S1	S2	1.1	1.2	1.3	1.4	1.5	2.1	2.2	2.3	2.4	2.5	3.1	3.2	3.3	3.4	3.5	4.1	4.2	4.3	4.4	4.5	5.1	5.2	5.3	5.4	5.5
Abdelmutti 2019	Quantitative descriptive study	Y	Y																Y	Y	CT	CT	Y					
Abdelrahim 2018	Qualitative	Y	Y	Y	Y	Y	Y	Y																				
Bricker 2020	Randomised controlled trial	Y	Y						Y	Y	Y	CT	Y															
Carroll 2019	Qunatitative non-randomised study	Y	Y											Y	Y	Y	CT	Y										
Charlot 2019	Mixed methods study	Y	Y																					Y	Y	CT	CT	CT
Cinciripini 2019	Quantitative non-randomised study	Y	Y											Y	Y	Y	Y	Y										
Conlon 2020	Qunatitative non-randomised study	Y	Y											Y	Y	Y	Y	Y										
Crawford 2019	RCT -not in paper but linked via code	Y	Y						CT	Y	Y	CT	Y															
Davidson 2017	Quantitative descriptive	Y	Y																CT	Y	CT	CT	Y					
Day 2020	Quantitative non-randomised study	Y	Y											Y	Y	Y	Y	Y										
Foshee 2017	Randomised controlled trial	Y	Y						N	CT	N	CT	CT															
Gali 2020	Quality Improvement Programme	N	N																									
Ghosh 2016	Randomised controlled trial	Y	Y						Y	Y	N	CT	Y															

Supplemental Table Quality Review of Studies

Guililani 2019	Quantitative non-randomised study	Y	Y											Y	Y	Y	CT	Y										
Krebs 2019	Randomised controlled trial	Y	Y						Y	Y	Y	CT	Y															
Ma 2016	QIP	N	N																									
McDonnell 2016	Mixed methods study	Y	Y																					Y	CT	CT	N	N
Miele 2018	Quantitative non randomised ?? CHANGE TO rct	Y	Y											Y	Y	Y	Y	Y										
Nolan 2019	QIP	N	N																									
Park 2020	Randomised controlled trial	Y	Y						Y	Y	Y	N	Y															
Phillips 2020	Quantitaive non-randomised study	Y	Y											Y	Y	Y	CT	Y										
Price 2017-randomisation in table	Quantitative andomized study	Y	Y						CT	Y	Y	CT	Y															
Ramsey 2020	Quantitaive non-randomised study	Y	Y											Y	Y	Y	CT	Y										
Rettig 2018	Randomised controlled trial	Y	Y						Y	CT	Y	CT	Y															
Schnoll 2019-randomised in paper	Quantitative randomized study	Y	Y						CT	Y	Y	CT	Y															
Simmons 2020	Randomised controlled trial	Y	Y						Y	Y	Y	N	Y															
Smaily 2021	Randomised controlled trial	Y	Y						CT	Y	Y	CT	CT															
Smith 2019	Mixed methods study	Y	Y																					Y	Y	CT	CT	CT
Y=Yes																												
N=No																												
CT = Can't tell																												
